# Increasing the production of (*R*)-3-hydroxybutyrate in recombinant *Escherichia coli* by improved cofactor supply

**DOI:** 10.1186/s12934-016-0490-y

**Published:** 2016-06-01

**Authors:** Mariel Perez-Zabaleta, Gustav Sjöberg, Mónica Guevara-Martínez, Johan Jarmander, Martin Gustavsson, Jorge Quillaguamán, Gen Larsson

**Affiliations:** Division of Industrial Biotechnology, School of Biotechnology, KTH Royal Institute of Technology, AlbaNova University Center, SE 106 91 Stockholm, Sweden; Center of Biotechnology, Faculty of Science and Technology, Universidad Mayor de San Simón, Cochabamba, Bolivia

**Keywords:** *Escherichia coli*, *Halomonas boliviensis*, (*R*)-3-hydroxybutyrate, Acetoacetyl-CoA reductase, NADPH, *zwf* overexpression, Glutamate, Nitrogen limitation

## Abstract

**Background:**

In a recently discovered microorganism, *Halomonas boliviensis,* polyhydroxybutyrate production was extensive and in contrast to other PHB producers, contained a set of alleles for the enzymes of this pathway. Also the monomer, (*R*)-3-hydroxybutyrate (3HB), possesses features that are interesting for commercial production, in particular the synthesis of fine chemicals with chiral specificity. Production with a halophilic organism is however not without serious drawbacks, wherefore it was desirable to introduce the 3HB pathway into *Escherichia coli.*

**Results:**

The production of 3HB is a two-step process where the acetoacetyl-CoA reductase was shown to accept both NADH and NADPH, but where the *V*_*max*_ for the latter was eight times higher. It was hypothesized that NADPH could be limiting production due to less abundance than NADH, and two strategies were employed to increase the availability; (1) glutamate was chosen as nitrogen source to minimize the NADPH consumption associated with ammonium salts and (2) glucose-6-phosphate dehydrogenase was overexpressed to improve NADPH production from the pentose phosphate pathway. Supplementation of glutamate during batch cultivation gave the highest specific productivity (q_3HB_ = 0.12 g g^−1^ h^−1^), while nitrogen depletion/*zwf* overexpression gave the highest yield (Y_3HB/CDW_ = 0.53 g g^−1^) and a 3HB concentration of 1 g L^−1^, which was 50 % higher than the reference. A nitrogen-limited fedbatch process gave a concentration of 12.7 g L^−1^ and a productivity of 0.42 g L^−1^ h^−1^, which is comparable to maximum values found in recombinant *E. coli*.

**Conclusions:**

Increased NADPH supply is a valuable tool to increase recombinant 3HB production in *E. coli*, and the inherent hydrolysis of CoA leads to a natural export of the product to the medium. Acetic acid production is still the dominating by-product and this needs attention in the future to increase the volumetric productivity further.

**Electronic supplementary material:**

The online version of this article (doi:10.1186/s12934-016-0490-y) contains supplementary material, which is available to authorized users.

## Background

Microorganisms are increasingly used for production of renewable chemicals and biomaterials [1]. One example is the polymer polyhydroxybutyrate (PHB), but also other commercially interesting polyhydroxyalkanoates have been investigated for use as bioplastics [2]. The monomer of PHB, (*R*)-3-hydroxybutyrate (3HB), has potential applications apart from polyester production, e.g. as an intermediate in the synthesis of antibiotics, vitamins, aromatic derivatives and pheromones [3–6]. Several of these applications require a specific enantiomer of 3HB, which makes microbial production highly interesting due to the stereospecificity of the involved enzymes.

*Halomonas boliviensis* is a halophilic bacterium that is known to accumulate PHB to over 80 % of its cell dry weight (CDW) and to achieve volumetric productivities of up to 1.1 g L^−1^ h^−1^ [7]. The genome of this microorganism has experienced significant horizontal gene transfer and has thus obtained a wide range of genes for carbohydrate metabolism, as well as for PHB production [8]. For the production of PHB three enzymes are needed and *H. boliviensis* possesses seven alleles of the β-ketothiolase, one allele of the acetoacetyl-CoA reductase and three alleles of the PHB synthase. These alleles are however scattered over the chromosome and are not organized into operons, as in e.g. *Cupriavidus necator* [9]. Due to the efficient production of PHB in *H. boliviensis*, this organism is an interesting source of enzymes for recombinant production of PHB as well as 3HB. The engineering of *H. boliviensis* is however considerably more difficult than common commercial organisms due to the lack of genetic tools and metabolic data. In addition, cultivation of *Halomonas* strains is associated with a need for high salt concentrations, making it unsuitable for use in commercial stainless steel bioreactors due to corrosive damage. We have thus chosen to express the genes for 3HB production from *H. boliviensis* in *Escherichia coli,* an organism that does not naturally produce PHB. From earlier experiments it was seen that *E. coli* most likely expresses the enzyme thioesterase II (*tesB*), which is capable of hydrolysis of (*R*)-3-hydroxybutyrate-CoA (3HB-CoA), which in turn leads to excretion of 3HB to the medium [10]. This is an added benefit with expression in *E. coli* since its results in a primary purification of the product.

**Fig. 1:**
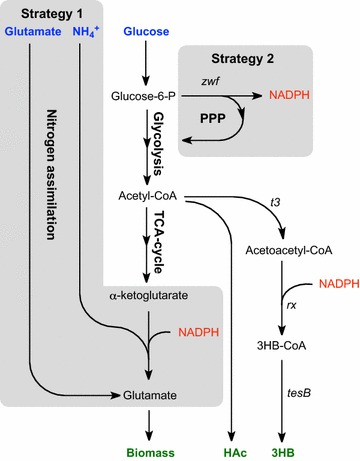
Simplified metabolic scheme showing the two strategies for increased NADPH availability in connection to the 3HB production pathway. The first strategy involves reducing the NADPH expenditure of the cell by providing glutamate as a second nitrogen source to ammonia, allowing a major NADPH sink to be bypassed. The second strategy involves overexpression of glucose-6-phosphate dehydrogenase (*zwf*) to redirect some of the glycolytic flux to the pentose phosphate pathway, increasing the amount of NADPH synthesized

The synthesis of 3HB is a two-step reaction. The enzyme β-ketothiolase first catalyzes the condensation of two molecules of acetyl-CoA to acetoacetyl-CoA where after the acetoacetyl-CoA reductase further catalyzes the formation of 3HB-CoA (Fig. 1). From the seven alleles of β-ketothiolase found in *H. boliviensis* the *t3* gene was initially selected due to its suggested sequence similarity with the thiolase of *C. necator*. Only one allele for the reductase was found in *Halomonas boliviensis* (*rx*), which was chosen but where the cofactor was unknown due to the recent discovery of the organism. Many acetoacetyl-CoA reductases involved in PHB formation are known to be NADPH-dependent but the acetoacetyl-CoA reductase obtained from *Allochromatium vinosum* is one exception [2] and the specific cofactor-dependence can thus not be presumed.

## Results and discussion

### Cofactor specificity of the *H. boliviensis* acetoacetyl-CoA reductase

**Fig. 2:**
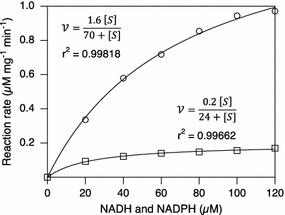
Kinetic model of His-tagged acetoacetyl-CoA reductase synthesized by gene *rx* that was obtained from—the *H. boliviensis.* Symbols refer to NADH (*squares*) and NADPH (*circles*). The reaction rates at different concentrations of NADH and NADPH, respectively, were curve fitted to the Michaelis–Menten equation to obtain the values of V_max_ and K_M_ for each cofactor

In order to determine the cofactor specificity of the reductase, the enzyme was produced in *E. coli* and purified using metal ion chromatography. The kinetic parameters of the reductase were subsequently analyzed using either NADH or NADPH as cofactors with acetoacetyl-CoA as substrate (Fig. 2). The values of the maximum rates (*V*_*max*_) were 1.6 μM mg^−1^ min^−1^ for NADPH and 0.2 μM mg^−1^ min^−1^ for NADH. Furthermore, the saturation constants (*K*_*M*_) were 70 and 24 μM, for NADPH and NADH, respectively (Table 1). From Fig. 2 and Table 1 it is evident that increasing the NADPH availability could potentially have a big impact on the conversion rate of acetoacetyl-CoA to 3HB-CoA, since the *V*_*max*_ for NADPH is eight times higher than for NADH. These results suggest that 3HB-CoA synthesis is adaptable to different conditions in *H. boliviensis*, i.e. that a basal production rate can be maintained using NADH also when NADPH concentrations are low, while it can be greatly increased whenever an excess of NADPH is available.

**Table 1:** Kinetic parameters of the acetoacetyl-CoA reductase from *H. boliviensis*

Cofactor	V_max_ (μmol mg^−1^ min^−1^)	K_M_ (μM)	k_cat_ (s^−1^)	k_cat_/K_M_ (s^−1^ μM^−1^)
NADPH	1.6 ± 0.2	70 ± 6	780 ± 100	11.1 ± 0.3
NADH	0.20 ± 0.02	24 ± 6	100 ± 7	4.2 ± 0.2

**Fig. 3:**
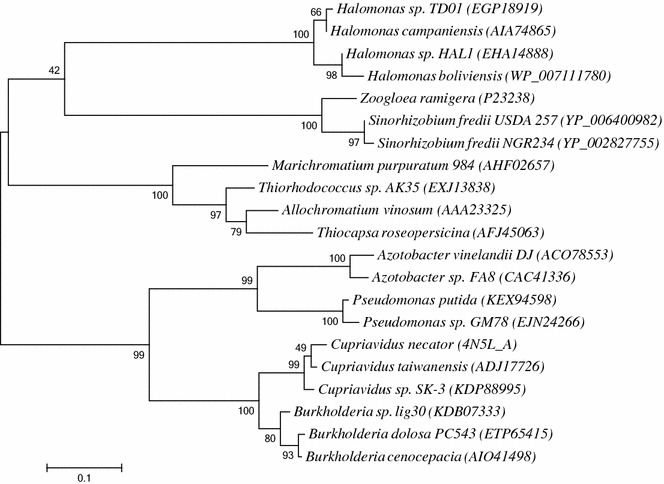
Phylogenetic tree of *Halomonas boliviensis* related acetoacetyl-CoA reductases. Phylogenetic relationship among enzymes was found by maximum-likelihood (ML) tree-building strategy. GenBank accession numbers are given in parenthesis

A phylogenetic study was conducted, comparing the *Halomonas* reductase gene (*rx*) to other reductase genes with known cofactor specificity. Figure 3 shows that *rx* is most closely related to reductases from other *Halomonas* species and a group containing the reductase from *Zoogloea ramigera,* which has been found to be NADPH dependent [11]. In a more distant branch, a third cluster is formed that contain a reductase from *A. vinosum* which is known to be NADH dependent [12]. The well-known NADPH dependent reductase in the *phbCAB* operon [9] from of *Cupriavidus necator* (previously known as *Ralstonia eutropha* and *Alcaligenes eutrophus*), is however one of the most distantly related reductases. From the phylogenetic tree (Fig. 3), it could be concluded that *rx* exhibited similarity to both NADPH and NADH dependent reductase genes. Taken together, it can be concluded that the *H. boliviensis* reductase (*rx*) accepts both NADPH and NADH as the reducing agent while there is a clear preference towards NADPH.

### Increasing NADPH availability

A critical step in recombinant production from inserts of whole new synthesis pathways is the supply and generation of cofactors which points to the acetoacetyl-CoA reductase catalyzed step as crucial. The availability of NADPH is restricted and we thus hypothesized that increased availability might improve the 3HB production. The first attempt to improve the cofactor availability was to reduce the amount of NADPH consumed by the cell which was done by shifting the nitrogen source from ammonium salt to glutamate (Fig. 1). This would bypass the first step in ammonia assimilation, where the cell spends one equivalent of NADPH when glutamate is produced from ammonia and α-ketoglutarate, leaving it available for 3HB-CoA production. We found however that the uptake of glutamate in *E. coli* is very slow, resulting in almost no growth if utilized as the only nitrogen source. A medium containing both ammonium and glutamate was therefore used. In the second attempt, the gene *zwf*, which encodes for the enzyme glucose-6-phosphate dehydrogenase catalyzing the first committed step in the pentose phosphate pathway (PPP), was overproduced (Fig. 1). This reaction sequence is the major cellular source of NADPH formation and *zwf* overexpression was previously described to improve cofactor balance however in PHB production [13].

Both NADPH-increasing strategies were first evaluated in bioreactors during batch conditions with unrestricted cell growth and at glucose excess. This leads to allosteric inhibition of citrate synthase by NADH, a reduced TCA activity and the formation of acetic acid due to the glycolytic overflow [14]. It was speculated that the introduction of the 3HB pathway would lead to a redirection of the carbon flux from acetic acid to this product. As shown in the first part of Table 2, the strain with access to glutamate produced 3HB at the highest specific rate i.e. q_3HB_ = 0.12 g g^−1^ h^−1^, while the strain overexpressing *zwf* gave the highest concentration i.e. 0.73 g L^−1^. Both strategies resulted in higher 3HB concentrations, higher yields and both improved specific and volumetric production rates as compared to the reference. It was further noted that the strain carrying the production plasmids as well as the additional plasmid for *zwf* overexpression did not suffer from the increased metabolic load and the specific growth rate remained high.

**Table 2:** Production of (*R*)-3-hydroxybutyrate by using strategies for increased NADPH availability

Process	Plasmids	Nitrogen source	Time (h)	CDW (g L^−1^)	Glucose consumed (g L^−1^)	3HB (g L^−1^)	μ (h^−1^)	r_3HB average_ (g L^−1^ h^−1^)	q_3HB average_ (mg g^−1^ h^−1^)	Y_3HB/Glc_ (g g^−1^)	Y_3HB/CDW_ (g g^−1^)	Reference
Unrestricted	pJBGT3RX	NH_4_^+^	5.5	4.18	8.90	0.51	0.73	0.093	68	0.057	0.12	[16]
Unrestricted	pJBGT3RX	NH_4_^+^ + glutamate	6.5	3.84	10.40	0.68	0.72	0.105	116	0.065	0.18	This study
Unrestricted	pJBGT3RX + pBADzwf	NH_4_^+^	7.0	3.69	10.01	0.73	0.68	0.104	75	0.073	0.20	This study
N-depletion	pJBGT3RX	NH_4_^+^	11.8	2.54	7.05	0.50	–	0.042	17	0.071	0.20	[15]
N-depletion	pJBGT3RX	NH_4_^+^ + glutamate	11.9	2.39	7.75	0.55	–	0.046	35	0.071	0.23	This study
N-depletion	pJBGT3RX + pBADzwf	NH_4_^+^	11.8	2.00	8.46	1.06	–	0.090	57	0.125	0.53	This study
N-Fedbatch	pJBGT3RX + pBADzwf	NH_4_^+^	30.0	34.80	98.10	12.70	–	0.423	14	0.129	0.36	This study

In the cases of nitrogen depletion and fedbatch, q_3HB_ are calculated for the respective limited/depleted phase only, and do not include the excess-nutrients phase. Process names refer to: Unrestricted, means that all nutrients were supplied in the medium as regular batch cultivation. N-depletion, cultivation with two phases, a short batch with all nutrients in excess and a nitrogen depletion phase. N-Fedbatch, cultivation with two phases, a repeated batch where the nutrients were added repeated times to kept in excess and a nitrogen limitation fedbatch phase

### Cultivations using nitrogen depletion for improved 3HB production

Since both strategies for improving the intracellular NADPH levels were promising, we hypothesized that the productivities, yields and titers from the batch cultivations could be further improved by depletion of nitrogen while glucose remained in excess. This is a well-known cellular strategy in wild type polyhydroxyalkanoate (PHA) producers where a specific limitation e.g. in nitrogen, controls the growth rate while the NADH overflow from a high glycolytic flux leads to TCA cycle restriction, thus promoting a redistribution of the carbon flux from cell growth to the PHA production.

**Fig. 4:**
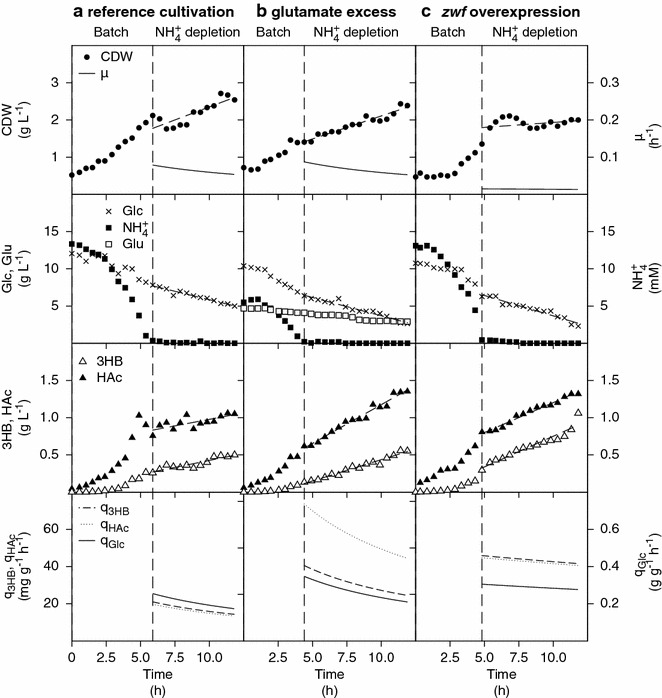
Production of (*R*)-3-hydroxybutyrate in *E. coli* AF1000 **a** pJBGT3RX, reference cultivation. **b** pJBGT3RX with excess of glutamate in the medium **c** pJBGT3RX pBADzwf, in batch mode during ammonium depletion. Symbols refer to cell dry weigh (CDW, *filled circles*), glucose (Glc, *crosses*), glutamate (Glu, *open squares*), ammonium (NH_4_^+^, *closed squares*), 3HB (*open triangles*) and acetic acid (HAc, *closed triangles*). The specific productivity of 3HB has been curve fitted to a 1st order polynomial. The *dashed line marks* the shift between batch cultivation and nitrogen depletion

The cultivations were performed in two phases: (1) a batch phase with all nutrients in excess followed by phase (2) i.e. nitrogen depletion but with all other nutrients present in excess. As shown in Fig. 4, the cell mass in phase 2 slightly increases in a linear fashion despite the lack of the major nitrogen substrate component, which is different from the case when glucose is depleted and the cell immediately enters into a stationary phase. In the figure is also seen that in spite of the ammonium depletion 3HB production continues while cell growth is radically reduced which confirms the presence of the metabolic regulation targeted above. In Fig. 4 is seen that the highest specific production rate of 3HB was observed with overexpression of *zwf* (q_3HB_ = 0.06 g g^−1^ h^−1^) and this led to a 50 % higher 3HB titer (1 g L^−1^) than in the reference cultivation. The reduced cell growth during the depletion phase results also in a substantially increase of the yield, Y_3HB/CDW_ and in the highest value achieved i.e. 0.53 g g^−1^ (Table 2). Since 3HB production kinetics follows a mixed growth pattern with production both during growth and depletion, nitrogen starvation can be an interesting strategy to pursue but to increase the productivity, the starvation should be preceded by a phase with maximized cell mass accumulation.

3HB is not the only product in the cultivations since acetic acid (HAc) is at all times formed and this route should obviously be avoided since it draws resources from the 3HB formation. Comparing the HAc production for the cultivations in Fig. 4, it is evident that both *zwf* overexpression and the use of a glutamate-supplemented medium led to increased HAc production. In the case of *zwf* overexpression, the specific production rate of HAc (q_HAc_) correlates well with the specific production rate (q_3HB_), which is in line with previous ammonium-depleted cultivations [15]. However, in the case of glutamate excess, HAc production seems not directly coupled to 3HB production and here we also find the highest HAc production.

Several factors might lead to restrictions in the 3HB production whereof one is a restricted hydrolysis of coenzyme A (CoA) and a reduced export of 3HB to the medium. Overexpression of thioesterase II (*tesB*) has been one method to overcome this supposed bottleneck and was previously used to increase 3HB production during recombinant 3HB production in *E. coli* but using the genes of *C. necator* [10]. However, *tesB* overexpression in our strain did not improve the 3HB production and resulted in impaired cell growth (Additional file 1: Table S1).

### Maximizing the 3HB overproduction by fedbatch cultivation

**Fig. 5:**
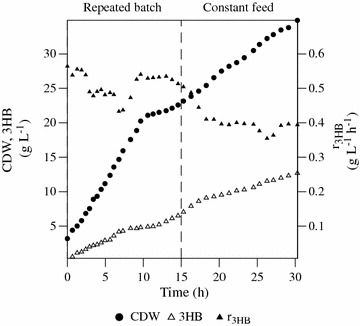
Production of (*R*)-3-hydroxybutyrate by *E. coli* AF1000 pJBGT3RX pBADzwf. Symbols refer to cell dry weigh (CDW, *filled circles*), 3HB (*open triangles*) and volumetric 3HB production rate (r_3HB_, *closed triangles*). The *dashed line marks* the shift between repeated batch and the fedbatch phase, respectively

The strategy of *zwf* overexpression seemed to be a suitable means to increase 3HB production in favour of the glutamate feed strategy, which increased the undesired formation of acetic acid (Fig. 4). Nitrogen-limited fedbatch cultivation was thus designed to obtain a higher volumetric productivity than possible in simple batch cultivations. The principle outline was a two-phase process where the first phase was designed for accumulation of cell mass under high glucose concentration followed by a fedbatch phase with nitrogen limitation but a continued high feed of glucose. The cultivation in phase 1 was started with repeated batches to avoid the osmotic effects and inhibitions of growth due to the high nutrient concentrations resulting from a single addition. Glucose and (NH_4_)_2_SO_4_ was thus added repeatedly giving a final cell density of 22 g L^−1^ (Fig. 5) without oxygen limitation. At 15 h, nitrogen was exhausted and a constant feed phase started where both glucose and (NH_4_)_2_SO_4_ was added. The glucose was kept in excess, i.e. above 10 g L^−1^ while the nitrogen was added to limit growth. During the first 10 h of the experiment, the cells grew with a high specific growth rate (μ), which was subsequently reduced (Fig. 5) and this was attributed to toxic effects of the accumulation of acetic acid.

The highest volumetric productivity of 3HB was observed in the repeated batch phase with a maximum value of 0.56 g L^−1^ h^−1^ (Fig. 5) but with an overall productivity of 0.42 g L^−1^ h^−1^ (Table 2). The final concentration was 12.7 g L^−1^ that was achieved after 30 h of cultivation. This value is higher than in previously published work on 3HB production by recombinant *E. coli* [15–17]. Using genes from *H. boliviensis*, Guevara et al. [15] and Jarmander et al. [16] obtained 4.1 g L^−1^ after 13 h and 1.9 g L^−1^ after 11 h of cultivation, respectively. Gao et al. [17] used genes from *C. necator* and achieved to a concentration of 12 g L^−1^ 3HB after 48 h of cultivation under a repetitive batch mode. Furthermore, the concentration and productivity of 3HB in this study are comparable to the work of Liu et al. where genes from *C. necator* were overexpressed as well as the *tesB* gene which was here necessary for increased extracellular production [10].

## Conclusions

Several factors influence the production of metabolites from transplantation of new pathways into microorganisms and one central point is the balancing of cofactors. The preferred cofactor of the reductase gene transplanted from *Halomonas boliviensis* was shown to be NADPH to which the access is limited in the cell in comparison to NADH. We thus hypothesized that this could constitute a bottleneck in 3HB production and increasing the supply would allow a higher production. This hypothesis was verified since the proposed strategies both lead to increased product formation and where the *zwf* overexpression strategy gave 50 % better results than the reference. The attempt to reduce NADPH consumption was not as successful due to the low assimilation rate of glutamate in *E. coli*. The high 3HB titer of 12.7 g L^−1^ and the volumetric productivity of 0.42 g L^−1^ h^−1^ during *zwf* overexpression are comparable to maximum values found in the literature with *E. coli* however based on genes from *C. necator* [10].

The productivity in *E. coli* so far is however only 30 % of the values in *H. boliviensis* and a main factor is the accumulation of acetic acid, which is still present in spite of the use of the wild type PHB strategy with glucose excess and nitrogen limitation to force the carbon flux to 3HB. This is undesired since it lowers the growth rate and captures the carbon aimed for the product. This points however also to the important fact that the 3HB precursor acetyl-CoA is present in abundance.

## Methods

### Strains and plasmids

The *E. coli* strain AF1000 (*MC4100*, *relA1*^+^) [18] was used for expression of the genes *rx*, *rxHis*_*6*_, *t3* and *zwf,* as well as for production of 3HB. The *E. coli* strain DH5α was used for all cloning procedures. The plasmid pJBGT3RX was used for 3HB production. It was constructed by SLIC insertion of *rx* and *t3* to pKM1D, a pACYC184-derived low copy number plasmid with *ori* p15A, a lacUV5 promoter, a multiple cloning site, the lacIq repressor and a chloramphenicol resistance gene (Additional file 1: Figure S1). The plasmid pBAD*zwf* for increasing intracellular NADPH concentrations was constructed from pBAD/HisC (Invitrogen), a plasmid with the pBR322 *ori*, the araBAD promoter and an ampicillin resistance gene. From DH5α, *zwf* was amplified by PCR with complementary tails to the NcoI and EcoRI sites of pBAD/HisC. The plasmid was digested with EcoRI and NcoI followed by SLIC assembly [19, 20] to *zwf*. The plasmid pTrc99RXHis_6_ for purifying *rxHis*_*6*_ was constructed from pTrc99A [21], a plasmid with pBR322 *ori*, the lacI repressor, the trc promoter and an ampicillin resistance gene. From pJBGT3RX, the gene *rx* was amplified by PCR with complementary tails to the NcoI site of pTrc99A. The plasmid was digested with NcoI followed by SLIC assembly [20] to *rx*.

### Growth medium and cultivation conditions

Cultivations were carried out in autoclaved minimal medium containing 7 g L^−1^ (NH_4_)_2_SO_4_, 1.6 g L^−1^ KH_2_PO_4_, 6.6 g L^−1^ Na_2_HPO_4_·2 H_2_O and 0.5 g L^−1^ (NH_4_)_2_-H-(citrate). After sterilization, antibiotics (ampicillin, 100 μg mL^−1^, for pTrc99A and pBAD; and chloramphenicol, 50 μg mL^−1^, for pJBG), glucose 10 g L^−1^, 1 mM MgSO_4_ and a 1000× trace elements solution (0.5 g L^−1^ CaCl_2_·2H_2_O, 16.7 g L^−1^ FeCl3·6H_2_O, 0.18 g L^−1^ ZnSO4·7H_2_O, 0.16 g L^−1^ CuSO_4_·5H_2_O, 0.11 g L^−1^ MnSO_4_·H_2_O, 0.18 gL^−1^ CoCl_2_·6H_2_O, 20.1 gL^−1^ Na-EDTA) were also added to the medium. In batch experiments, 10 L stirred tank reactor was used with 7 L of cultivation medium. The cultivation temperature was 37 °C and the pH were kept at 7 using 3 M NH_4_OH. The cultivations were performed until the glucose was depleted from the medium.

For ammonia depleted experiments, the concentration of (NH_4_)_2_SO_4_ was reduced to 1 g L^−1^ and (NH_4_)_2_-H-(citrate) was substituted with 0.65 g L^−1^ Na_3_-(citrate). Titration was in those cases carried out with 3 M NaOH. In the glutamate supplemented cultivations, glutamate was added to a final concentration of 4.5 g L^−1^.

Fedbatch fermentation was performed in a 15 L bioreactor containing initially 8 L of minimal medium. The fermentation process was started with a repeated batch phase followed by a constant feed of glucose and (NH_4_)_2_SO_4_, where the latter was the limiting substrate. In the repeated batch phase, glucose was added repeatedly in order to keep the concentration above 10 g L^−1^. The initial concentration of (NH_4_)_2_SO_4_ was 7 g L^−1^, when ammonium was depleted, as indicated by an increase in DOT, another 7 g L^−1^ was added to allow high cell density. When the ammonium was depleted for a second time, the constant feed started. The pH was adjusted using 9 M NaOH solution.

AF1000 pJBGT3RX or AF1000 pJBGT3RX pBADzwf were inoculated from a glycerol stock stored at −80 °C to shake flasks containing 500 ml cultivation medium. The cells were cultivated over night at 37 °C and 180 rpm and then used to inoculate the bioreactor. Induction was performed with 200 µM IPTG (for pJBGT3RX) and 0.002 % (w/w) L-arabinose (for pBADzwf) when the optical density at 600 nm (OD_600_) reached 0.2. Cultivations were performed aerobically and the dissolved oxygen was maintained above 30 % by manually increasing airflow and stirring speed. Antifoam was added as required.

### Analysis of cultivation samples

The OD_600_ was monitored in a spectrophotometer (Genesys 20, Thermo scientific) after dilution to an OD_600_ of approximately 0.1 in saline solution (0.9 %, w/v NaCl). CDW was determined, in triplicate, by taking 5 ml samples into pre-weighed dry glass tubes, which were centrifuged at 2000*g* for 5 min, followed by re-suspension of the pellet in 5 ml saline solution and a second centrifugation at 2000*g* for 5 min. The resulting cell pellets were dried over night at 105 °C and weighed the next morning. The supernatant of the CDW sample was filtered (VWR collection, 0.45 μm) and stored at −20 °C until analysis. These samples were used to measure the concentration of 3HB and HAc, using the enzymatic kits Megazyme D-3-hydroxybutyrate Assay (Cat No. K-DBHA) and Boehringer Mannheim (Cat No.10148261035) respectively. To determine the concentration of glucose, glutamate and nitrogen, samples of 2 ml cell culture were rapidly taken into pre-weighed tubes containing 2 ml of cold 0.13 M perchloric acid and then centrifuged at 2000*g* for 10 min. 3.5 ml of the supernatant was neutralized with 75 μL of cold K_2_CO_3_ (500 g L^−1^) and put on ice for 15 min to allow precipitation of salts. The sample was centrifuged again at 2000*g* for 5 min. Supernatants for analysis of glucose and ammonia were filtered (VWR collection, 0.45 μm) and stored at −20 °C until analysis. Ammonia and glucose concentrations were determined with the enzymatic kits Megazyme ammonia assay kit rapid (Cat No. K-AMIAR) and Boehringer Mannheim (Cat No. 10716251035) respectively. Glutamate concentrations were determined with BioProfile Flex Analyzer, Nova Biomedical. SDS-PAGE and western blot were performed as described previously [22] to confirm the presence of the enzymes coded for by the *rx* and *zwf* genes.

### Purification of recombinant acetoacetyl-CoA reductase

To study the cofactor dependence of *rx*, a glycerol stock stored at −80 °C of *E. coli* AF1000 pTrc99RX, containing *rx* with a His-tag, was inoculated to 500 ml minimal medium in a 5 L baffled shake flask. The cells were induced at OD 0.2 with 200 μM IPTG and cultivated at 37 °C and 180 rpm. At approximately OD 3, the cell culture was centrifuged and re-suspended in IMAC washing buffer (20 mM sodium phosphate, 0.5 M NaCl, 10 mM imidazole, pH 7.4) at 4 °C. For each gram of wet cell weight, 10 ml of buffer was added. The temperature was maintained at 4 °C throughout the purification. The cells were disrupted by French Press (French Pressure cell press, SLM Instruments) at 800 bar. The cell debris was then removed by centrifugation at 10.000*g* for 60 min. The enzyme was purified on a 5 ml HiTrap IMAC FF column charged with Ni^2+^ (GE Healthcare Life Sciences) according to the manufacturers instructions. The column was equilibrated with 10 column volumes of binding buffer (20 mM sodium phosphate, 0.5 M NaCl, 20 mM imidazole) at a flow of 5 ml min^−1^. The sample was thereafter loaded at 1 ml min^−1^. The column was washed with binding buffer at 5 ml min^−1^ until the absorbance at 280 nm reached a steady base-line. A step elution (20 mM sodium phosphate, 0.5 M NaCl, 500 mM imidazole) was done and a single fraction was collected. The eluted fraction was buffer changed to 125 mM Tris–HCl on a PD10 desalting column (GE Healthcare Life Sciences) according to the manufacturers instructions. The enzyme concentration was determined spectrophotometrically at 280 nm. Finally, kinetic analysis was performed immediately after the concentration determination.

### Kinetic characterization of the acetoacetyl-CoA reductase

The activity of the acetoacetyl-CoA reductase was assayed spectrophotometrically (Cary 50 Bio UV visible) at 340 nm and 25 °C. All reactions were performed with 100 µl enzyme solution and 20 µM of acetoacetyl-CoA. The final volume was 1 ml and 125 mM Tris–HCl (pH 8.0) was used as a buffer. The indicated concentrations (Fig. 2) of NADPH or NADH were used [23].

### Evolutionary and primary sequence analyses of acetoacetyl-CoA reductase

In order to study the cofactor dependence of the acetoacetyl-CoA reductase (*rx*) from *H. boliviensis*, its phylogenetic relatedness to enzymes with known specificity was studied. Protein sequences for acetoacetyl-CoA reductases from *H. boliviensis*, *Zoogloea ramigera*, *Allochromatium vinosum*, *Azotobacter vinelandii* and *Cupriavidus necator* were aligned using the Muscle program included in the MEGA 6 software package. To improve the bootstrap values between groups, sequences with high homology to the original enzymes were also included in the alignment. Phylogenetic relationship among enzymes was found by maximum-likelihood (ML) tree-building strategy, and was evaluated using the bootstrap method with 1000 bootstrap replications. The phylogenetic tree for reductases was constructed using a WAG with frequencies (+F) model, with uniform mutation rates among amino acid sites and complete deletion of gaps and missing data.

## Additional file

10.1186/s12934-016-0490-y Genetic map of pJBGT3RX. **Table S1.** Comparison of (*R*)-3-hydroxybutyrate production with and without *tesB* overexpression.

## Abbreviations

3HB: (*R*)-3-hydroxybutyrate
PHB: poly (3-hydroxybutyrate)
HAc: acetic acid
3HB-CoA: (*R*)-3-hydroxybutyrate-CoA
PPP: pentose phosphate pathway
OD: optical density
CDW: cell dry weight
Glc: glucose
Glu: glutamate
IPTG: isopropyl β-d-thiogalactoside
Y_3HB/CDW_: yield of (*R*)-3-hydroxybutyrate respect to cell dry weight
q_HAc_: specific production rate of acetic acid
q_3HB_: specific production rate of (*R*)-3-hydroxybutyrate
r_3HB_: volumetric (*R*)-3-hydroxybutyrate production rate
K_M_: saturation constant
k_cat_: rate constant
V_max_: maximum rate
DOT: dissolved oxygen tension
IMAC: immobilized metal affinity chromatography
PCR: polymerase chain reaction
SLIC: sequence and ligation independent cloning
SDS-PAGE: sodium dodecyl sulfate polyacrylamide gel
ML: maximum-likelihood
+F: frequencies
