# Supplementary material for: Increasing the production of (R)-3-hydroxybutyrate in recombinant Escherichia coli by improved cofactor supply
Source: Microb Cell Fact. 2016 Jun 1;15:91. doi: 10.1186/s12934-016-0490-y (PMC4888404; doi:10.1186/s12934-016-0490-y)
Supplement: Supplementary file 1 — 10.1186/s12934-016-0490-y Genetic map of pJBGT3RX. Table S1. Comparison of (R)-3-hydroxybutyrate production with and without tesB overexpression. [file 12934_2016_490_MOESM1_ESM.docx]

**Supporting information**

**Figure S1. Genetic map of pJBGT3RX.** t3 and rx represent the DNA fragment of β-ketothiolase and acetoacetyl-CoA reductase from *H. boliviensis*, respectively.

**
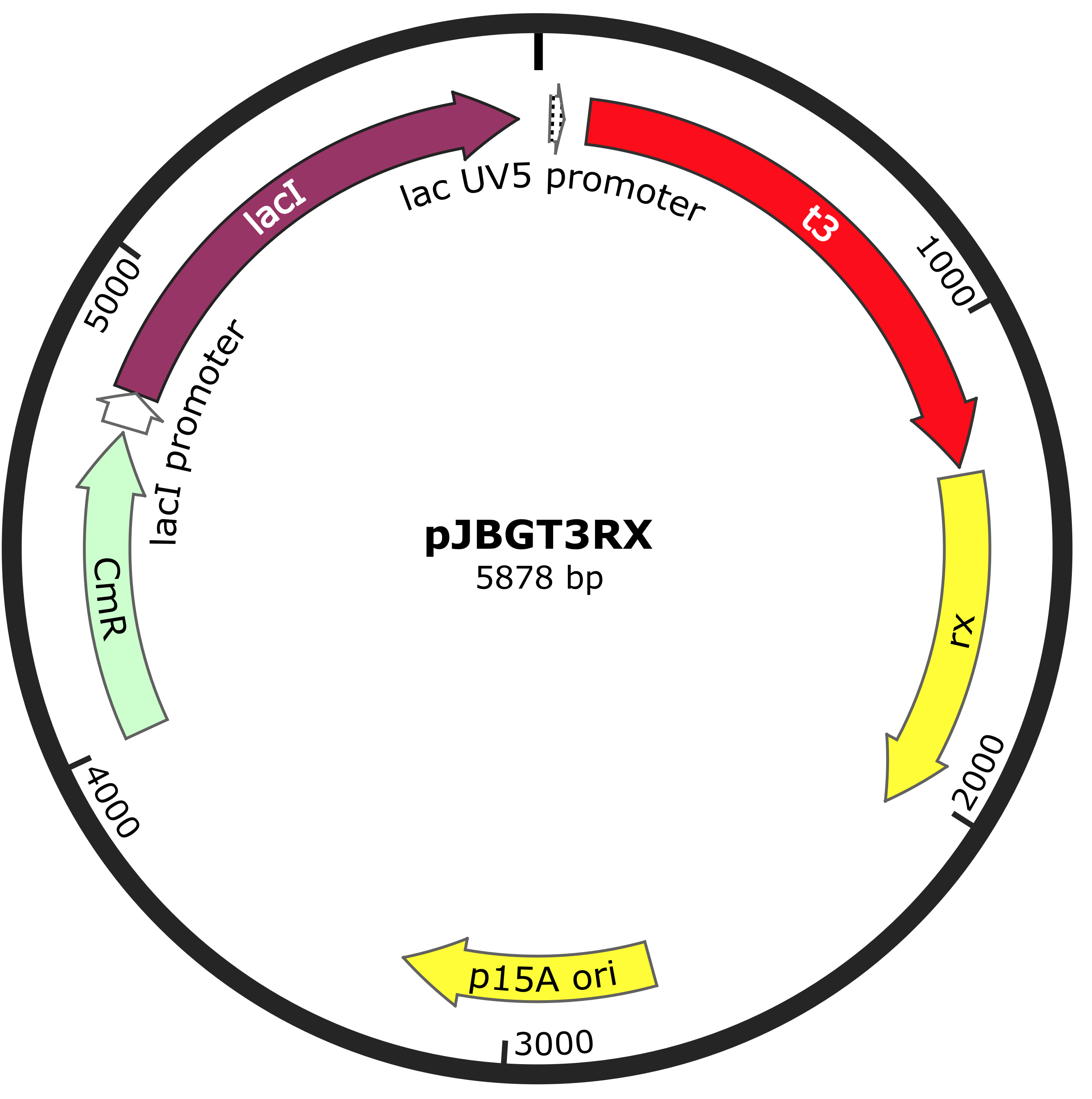
**

**Table S1. Comparison of (*R*)-3-hydroxybutyrate production with and without *tesB* overexpression.** Experiments were performed in shake flask, production was induced at OD_600_ = 0.2 with 200 uM IPTG.

| **Overexpression *tesB*** | **Growth rate (h^-1^)** | **Final OD_600_** | **3HB (g L^-1^)** | **Y_3HB/OD_**  **(g L^-1^ OD_600_^-1^)** |
| --- | --- | --- | --- | --- |
| - | 0.74 | 2.1 | 0.13 | 0.06 |
| + | 0.64 | 2 | 0.04 | 0.02 |
